# Change in singing behavior of humpback whales caused by shipping noise

**DOI:** 10.1371/journal.pone.0204112

**Published:** 2018-10-24

**Authors:** Koki Tsujii, Tomonari Akamatsu, Ryosuke Okamoto, Kyoichi Mori, Yoko Mitani, Naoya Umeda

**Affiliations:** 1 Ogasawara Whale Watching Association, Chichi-jima, Ogasawara-mura, Tokyo, Japan; 2 Graduate School of Environmental Science, Hokkaido University, Hakodate, Hokkaido, Japan; 3 National Research Institute of Fisheries Science, Japan Fisheries Research and Education Agency, Fukuura, Kanagawa, Japan; 4 Teikyo University of Science, Uenohara, Yamanashi, Japan; 5 Field Science Center for Northern Biosphere, Hokkaido University, Hakodate, Hokkaido, Japan; 6 Osaka University, Suita, Osaka, Japan; Sanya Institute of Deep-sea Science and Engineering Chinese Academy of Sciences, CHINA

## Abstract

Reactions of singing behavior of individual humpback whales (*Megaptera novaeangliae*) to a specific shipping noise were examined. Two autonomous recorders separated by 3.0 km were used for the acoustic monitoring of each individual song sequence. A passenger-cargo liner was operated once per day, and other large ship noise was excluded given the remote location of the Ogasawara Islands, 1000 km south of Tokyo. In total, locations of between 26 and 27 singers were measured acoustically using time arrival difference at both stereo recorders on the ship presence and absence days, respectively. Source level of the ship (157 dB rms re 1μPa) was measured separately in deep water. Fewer whales sang nearby, within 500 m, of the shipping lane. Humpback whales reduced sound production after the ship passed, when the minimum distance to the whale from the ship trajectory was 1200 m. In the Ogasawara water, humpback whales seemed to stop singing temporarily rather than modifying sound characteristics of their song such as through frequency shifting or source level elevation. This could be a cost effective adaptation because the propagation loss at 500 m from the sound source is as high as 54 dB. The focal ship was 500 m away within several minutes. Responses may differ where ship traffic is heavy, because avoiding an approaching ship may be difficult when many sound sources exist.

## Introduction

Ocean noise caused by human activities has been rapidly increasing in recent years. Andrew et al. [1] indicated that low-frequency noise levels in the ocean increased by 10 dB between 1960s and 1990s. Moreover, McDonald et al. [2] suggested that average noise levels would increase by 2.5–3 dB per decade. The sources of low-frequency noise are seismic explosion, transportation, harvesting renewable energy, and military sonar. Above all, the main noise source is presumed to be from commercial ships [1–3].

There has been concern about the effect of anthropogenic noise on marine creatures, including fishes and marine mammals (e.g., [4–7]). Specifically, its effect on cetaceans (whales, dolphins, and porpoises) is considered to be profound as cetaceans use sound to communicate, navigate, mate and search for prey under the water [8, 9]. Among the reported effects of shipping noise on cetaceans are stress increase [10], change in feeding behavior [11, 12], decrease in phonating individuals [13, 14], increase or decrease in sound production [15–17], and frequency or intensity modulation of their vocalizations [18, 19]. The results vary within and among species. Location- and species-specific reactions of baleen whales should be evaluated. Baleen whales (Mysticeti) are considered to be sensitive to low-frequency sounds such as shipping noise since they use low-frequency calls for communication [20, 21]. Therefore, evaluating how baleen whales respond to shipping noise while simultaneously measuring noise exposure levels is imperative.

In the Ogasawara Islands, which are located 1000 km south of Tokyo, Japan, humpback whales (*Megaptera novaeangliae*) of the western North Pacific (Asian) stock aggregate for breeding during winter months [22–24]. The number of humpback whales of the Asian population, which breed in the Ogasawara Islands, Okinawa, and the Philippines, is estimated to be from 938 to 1,107 [25]. Male humpback whales produce a “song” that is a complex vocal session composed of multiple sounds named “units”; a series of units is called a “phrase” and a sequence of phrases is called a “theme” in the breeding season [26–28]. The fundamental frequency of units is from tens of Hz to 4 kHz and higher harmonics reach 24 kHz [9, 29, 30]. The source level of song estimated in the previous report ranged from 151 dB to 173 dB rms re 1μPa [29]. Previous research hypothesized that a song may have numerous functions such as a display to attract females to individual singers, a signal to convey status or position among males, an organizer of males in the breeding ground, and/or as part of a lekking system by attracting females to a male aggregation [28, 31–34]. Even though the functions remain unclear, singing behavior is an appropriate focus for observing the noise effect because of its important role in the winter breeding season. Therefore, shipping noise may disturb the singing behavior of whales and could impact their reproduction. The previous studies reported that the negative effect on singing activity and the shortening of song duration by boat traffic [14, 35]. However, the information about the reaction of singing whales to the regular commercial ships is lack.

Passive acoustic monitoring has been widely conducted to monitor vocalization behavior of marine mammals. Characteristics of marine mammal sounds such as frequency, duration, and source level are species-specific [36]. By using a fixed hydrophone system, long-term monitoring of the presence of a target species is possible (e.g., [37, 38]). Additionally, we can distinguish individuals and identify their locations if we use multiple hydrophones [39, 40].

In this study, we focused on a passenger-cargo ship’s noise and the singing behavior of humpback whales in the Ogasawara Islands. There are two advantages to this study. First, because of the remoteness of the island, the noise source was singular and could be identified. Second, there was a stable aggregation of humpback whales in this area.

## Materials and methods

### Ethics statement

This study was conducted in the public waters. Permits and approvals for the deployment of recorders in the Ogasawara waters were obtained from Ogasawara Fisheries Cooperative Association on condition that the recorders do not prevent the ship navigation and are recovered after the end of the research (S1 File). Passive acoustic monitoring conducted in this study was a non-invasive method that involved no contact with the animals. Endangered or protected species were involved in this study.

### Source characteristics and recording systems

Sound source characteristics of the focal passenger-cargo liner with twin screw propellers were measured previously [41]. The ship has a 57.0 m length, a 12.0 m width, a 3.4 m draft, and 453 tons. The source level was 157 dB re 1μPa rms. Dominant energy was concentrated at 54 Hz when the twin screw propellers rotated at the usual navigation speed, which was up to 4.5 rotations per second. Following the ISO/DIS 16554.3 standard protocol [42], the sound measurement was conducted with a measuring boat while the target ship was cruising in front of the measuring boat. This measurement was carried out in deep water separately from the current experiment.

**Fig 1:**
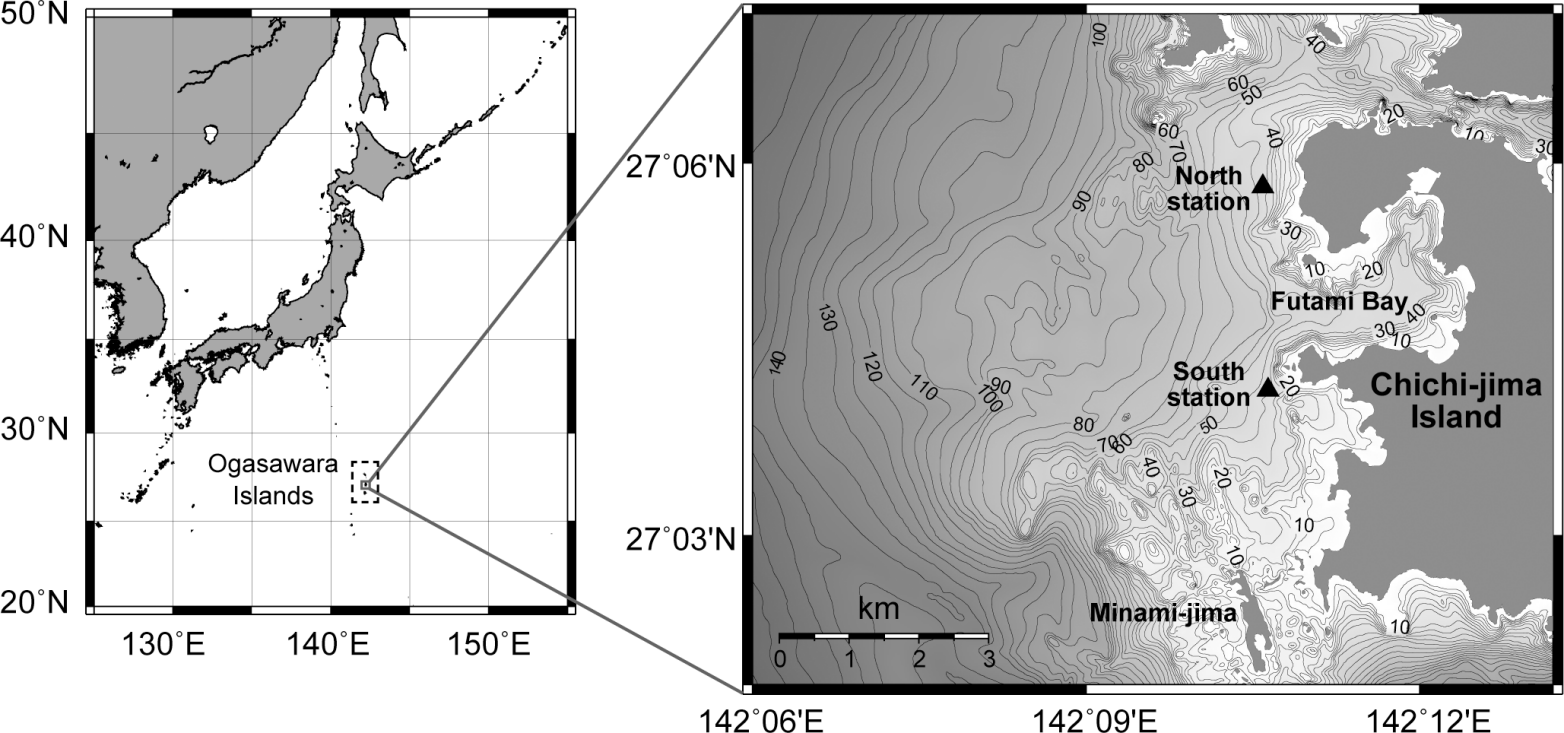
The study site, Chichi-jima Island, Ogasawara, Tokyo, Japan. The Ogasawara Islands are a UNESCO world natural heritage site known to be a major breeding ground of humpback whales of the western North Pacific (Asian) Stock. The north and south stations were deployed at about 40 m depth off the coast of Chichi-jima (black triangle). The recorders were suspended at a depth of 20 m using buoys, with a marker buoy situated at 4 m below the water surface. The bathymetry data was based on the M7023 digital bathymetry chart provided by Japan Hydrographic Association.

A stereo autonomous underwater recorder (AUSOMS-mini stereo, AquaSound Inc., Kobe, Japan) was used for underwater monitoring. It has two hydrophones at both ends and can record for up to 21 days continuously using two UM1 batteries and a 32 GB micro SD card. The recording period was constrained by the battery power but not by the memory size. Because we intended to perform continuous long-term recording, a compressed data format (mp3, 128 kbps) was used to reduce memory consumption. This compressed format allowed recording at a frequency of up to 17 kHz. Two AUSOMS-mini stereos were deployed at 27°05'48.43"N, 142°10'35.48"E and 27°04'10.40"N, 142°10'38.30"E, respectively, off the west side of Chichi-jima Island, Ogasawara, Japan, from the middle of February to early May 2017 (Fig 1). Recorders were exchanged every two or three weeks before recording was stopped. Using a buoy system, the recorder was fixed horizontally in the middle of the water column at 20 m where the water depth was 40 m so that the sound source bearing angles from both recorders could be calculated. As shown in the S1 Appendix, clocks of two recorders were synchronized at the beginning and the end of the recording that enabled measurement of the third bearing angle from the middle point of the two recorders.

### Detections of song units

Acoustic characteristics of each unit such as the received power spectrum level, minimum, maximum frequencies, and duration of units in a song sequence were calculated using custom made software written on MATLAB (MathWorks, Natick, MA, USA). Detailed signal processing to detect song units are described in the S1 Appendix. A brief description of the algorithm is as follows. Local peaks of power spectrum were calculated every 92.8 ms (4096-point FFT, 50% overlap, blackman window). Nearest neighbors of local peaks were connected to extract the contour of units in the spectrogram. Isolated local peaks, i.e., those without any other local peaks nearby, were eliminated in the denoising procedure. Absolute time, minimum and maximum frequencies, and maximum spectrum level in the contour were calculated unit by unit. In addition, sound source bearing angles from the mid-point of two autonomous recorders were calculated precisely by the time shift of a visual image of contour by synchronized two recorder clocks.

### Calculation of singer’s location

**Fig 2:**
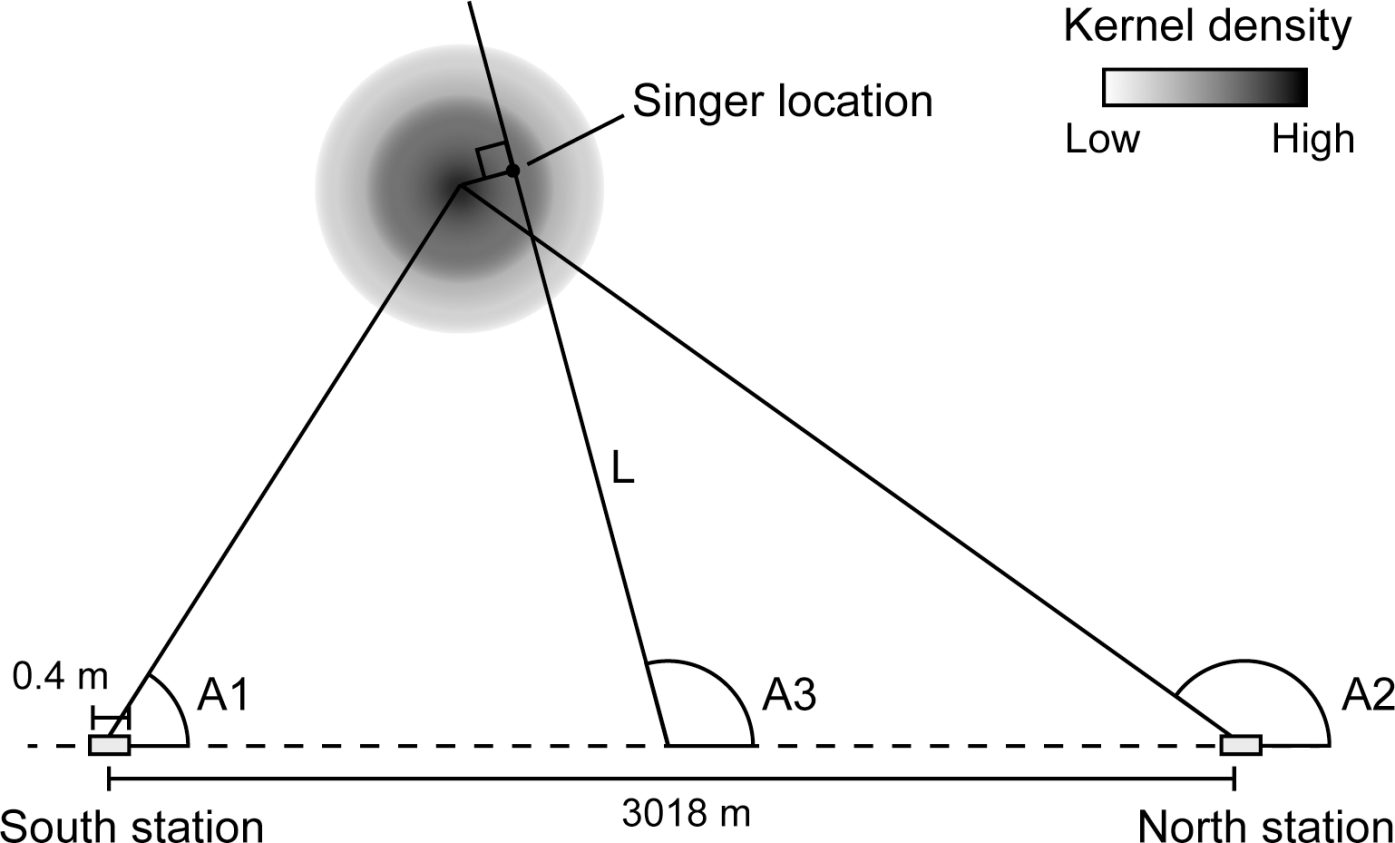
Calculation of singer’s location. Using two bearing angles from two recorders A1 and A2, the location of each unit could be calculated by triangulations. The bearing angle measured by the time shift of unit contour between two recorders (A3) is more accurate. The location of the singer was estimated as the cross point at the perpendicular line from the highest kernel density to the third bearing angle line (L).

Time arrival difference of the identical unit between two autonomous recorders was used to calculate the bearing angle from the middle point of the array. In addition, sound source bearing angles at each recorder measured by the cross correlation between stereo hydrophone recordings were used (Fig 2). Theoretically, a two-dimensional location can be calculated when two bearing angles from two locations are available. However, the resolution of the sound source bearing angles from the stereo recorder was limited because of a short baseline (40 cm) at the 44.1 kHz sampling frequency. This scattered the locations of calculated positions of song units from a single whale. The bearing angle calculation and triangulation were conducted every 9 seconds using custom-made software written on MATLAB. Error values due to the limited time resolution of the recorder, which gave locations out of the observable range, were eliminated. A kernel density estimation of all positions was depicted every 30 minutes. The 30-minute period from 7:30 to 8:00 am was sufficient to cover a test period when the target ship passed by the test area to calculate the location of singers close to the cruise ship line, as shown in the next section. The highest kernel density location is considered to represent the location of the singer. Appearance of multiple local peaks of kernel density was considered to be the case when two or more singers sing simultaneously. The individual closest to the ship trajectory of the ship was selected for further calculation. The bearing angles were also calculated by the time arrival difference between two time-synchronized autonomous recorders that are more accurate that the stereo recorder cross correlation. If several singers were identified from 7:30 to 8:00 am, detected units were separated into individuals according to the bearing angles. A cross point of the perpendicular line from the highest density of the kernel to the bearing line from the middle point of two recorders was defined as the location of the singer. At that time, the mean of bearing angles in 30 minutes was used.

### Data selection

Once the location of each singer had been identified, sound characteristics of the song sequence including during ship operation were selected. The ship departed at 7:30 am and passed the focal observation area between 7:40 and 7:50 am. The closest timing and distance of the ship to the whale was measured using the acoustically identified location of the whale and the GPS log of the target ship. The test period was defined as the 5 minutes before and after the closest approach time of the target ship. Because of the slight change in ship operation, the start time of the test period ranged from 7:33 am to 7:50 am, which was identified by daily observations. A “Cooling interval” was defined as the time intervals between the test and control periods. Pre- and post-control periods were selected as 7:15–7:25 and 8:05–8:15 when the test period was normally scheduled, which left a 15 minutes cooling interval after the ship passed by. The time of the control period shifted accordingly to the time schedule change of the ship. During a cooling interval of 15 minutes, the subject ship moved 8.5–10 km and left the area used during the test period. Assuming spherical sound propagation of the ship noise having a source level of 157 dB, the received level in the test area after the ship left the area was estimated as 77–78 dB, which is comparable to or lower than the background natural noise level. The ship was not operated daily due to bad weather or a scheduled break. The ship trajectory on February 20, 2017 was used as the model course to calculate the virtual distance from the singer to the ship trajectory in the days without ship operating, since this day had the most on-schedule ship among the days that we used in analysis. The maximum difference among the ship trajectories was 1.6 km at 142°09' E when the ship usually changed the direction to south.

Sound characteristics of the north station recording were used for the analysis. Since the south station is close to the ship trajectory, noise contamination of the focal ship was heard although the dominant frequency of the ship and the song differed. The north station is 3.0 km away from the south station and the sound propagation loss from the ship could be −70 dB if sound propagates spherically. Recordings of the north station were occasionally affected by a small fishing boat passing nearby. When contaminated by these noises during test or control periods, the whole acoustic data for that day were not used in further analysis. Furthermore, if the bearing of some units could not be precisely calculated because of the masking by the target ship noise in the recording data of the south station, results of detection were corrected manually using Adobe Audition CC (Adobe System Inc., San Jose, CA, USA).

### Statistical analyses

To examine whether the distribution of singing whales differed in the presence or absence of the target ship, a Kolmogorov–Smirnov test was performed on the two populations. In this test, the calculated closest distance from observed whales to the target ship was used in the comparison. We also tested the difference of singing behavior of whales between before and after the ship passed by the test area using a Wilcoxon signed rank test. The number of received units, maximum and minimum frequencies, received sound level, and duration of units were used as the parameters of singing behavior. In these analyses, the level of statistical significance was less than 5% (*P* < 0.05). All statistical analyses were conducted in R version 3.3.0 [43].

## Results

After the data selection, 1–3 singers per day and 26 singers in total were identified when the ship was present, although it could be that the same individuals were detected on different days. For comparison, 1–3 singers per day and 27 singers in total were identified without the presence of the ship during same time periods in a day (Table 1). Here, we obtained two sets of singing behavior of humpback whales within a known distance from the subject ship when the ship was on its closest approach to them (see S1–S5 Tables).

**Table 1:** The number of singers extracted from 6:55 to 8:25 on each day.

**Month**	Day																														
	**1**	**2**	**3**	**4**	**5**	**6**	**7**	**8**	**9**	**10**	**11**	**12**	**13**	**14**	**15**	**16**	**17**	**18**	**19**	**20**	**21**	**22**	**23**	**24**	**25**	**26**	**27**	**28**	**29**	**30**	**31**
**Feb-17**	-	-	-	-	-	-	-	-	-	-	-	-	-	NA	5	4*	NA	2	3*	4*	3	2*	3	2	3*	2*	4	NA*	4		
**Mar-17**	4	3	3*	4*	3	3*	2	3	3*	2*	2	3*	4	4	2*	4*	2	NA*	4	2	3*	3*	3	1*	3*	3	4	3	3*	2	2*
**Apr-17**	3*	4	2	3*	NA*	3	4*	2*	2	2	1*	2*	1	2*	1*	1	2	2*	2*	1	NA*	NA*	NA	NA	NA*	NA*	NA	NA*	NA*	NA*	
**May-17**	NA	NA*	NA*	NA	NA*	NA*	NA	NA*	NA	NA	-	-	-	-	-	-	-	-	-	-	-	-	-	-	-	-	-	-	-	-	-

The first deployment day of two recorders was on February 14, 2017, and the last recovery day was May 10, 2017. Grey painted days (35 days) were used in the analysis. The days that recording data had more than 4 singers or the other ship noises during the analysis periods were not used. NA indicates the days that recording data were not available because of exchanges of recorders or the recording failure. Asterisk shows the days that the target ship was operated at 7:30 am.

**Fig 3:**
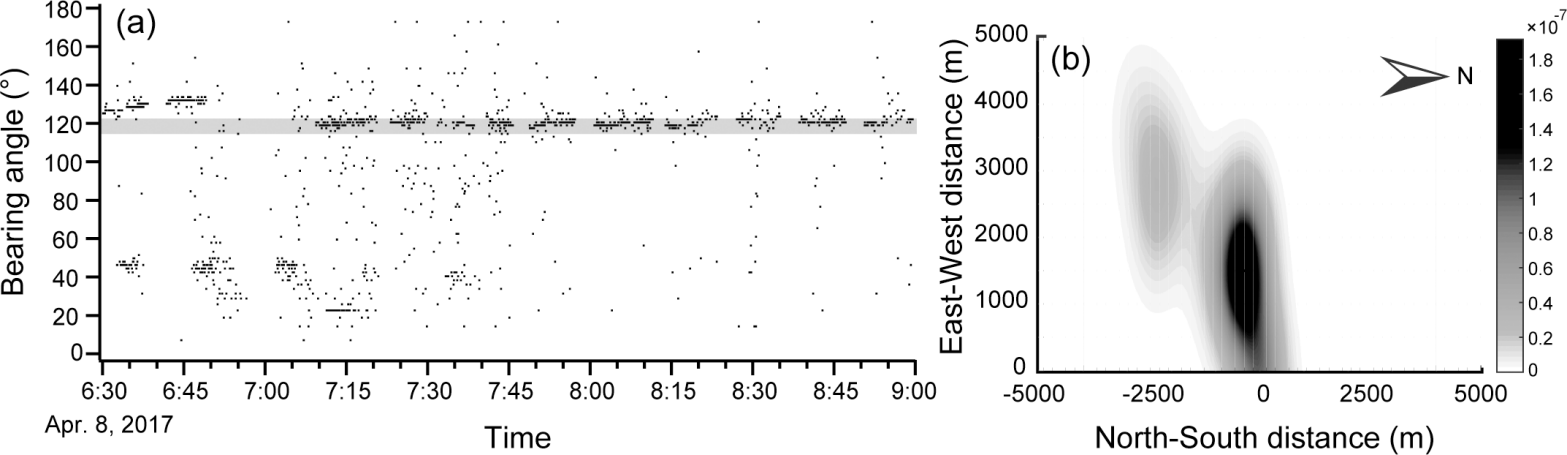
**(a) Sound source bearing angle changes according to the time and (b) the kernel density estimation of a singer during 30 minutes.** Both examples show that the individual singer did not move extensively during 30 minutes. The singer tended to be positioned in a specific location and singing was prolonged. Gray zone in (a) show mean and standard deviation of bearing angle from 7:30 to 8:00 am. The dots in (a) show the detected units. Y-axis in (a) shows the bearing angle to the detected sounds from the middle point between two recorders. Y-axis and X-axis in (b) show the East-West and North-South distance from the middle point between two recorders, respectively. The color in (b) indicates the intensity of time that a singer is predicted to be located. A letter “N” in (b) shows that the direction of the point of the arrow indicates north.

The bearing angle from the middle point of the recording stations of an individual singer did not change in a short time (Fig 3A). The mean (± standard deviation) of the bearing angle in the 30 minutes from 7:30 to 8:00 am, shown by the gray-painted area in Fig 3A, was 119.8 ± 3.2 degrees for this individual. The average of the bearing angle change was 0.4 ±1.9 degrees per minute. Including all individuals, i.e., 53 (26 + 27), the standard deviation of the bearing angle within 30 minutes was 3.3% of the average value of the bearing angle. This is also supported by the kernel density map of an individual singer during 30 minutes (Fig 3B), which shows the calculated locations of the singer were confined to a limited area. These results show that the singer did not move quickly when it was singing.

**Fig 4:**
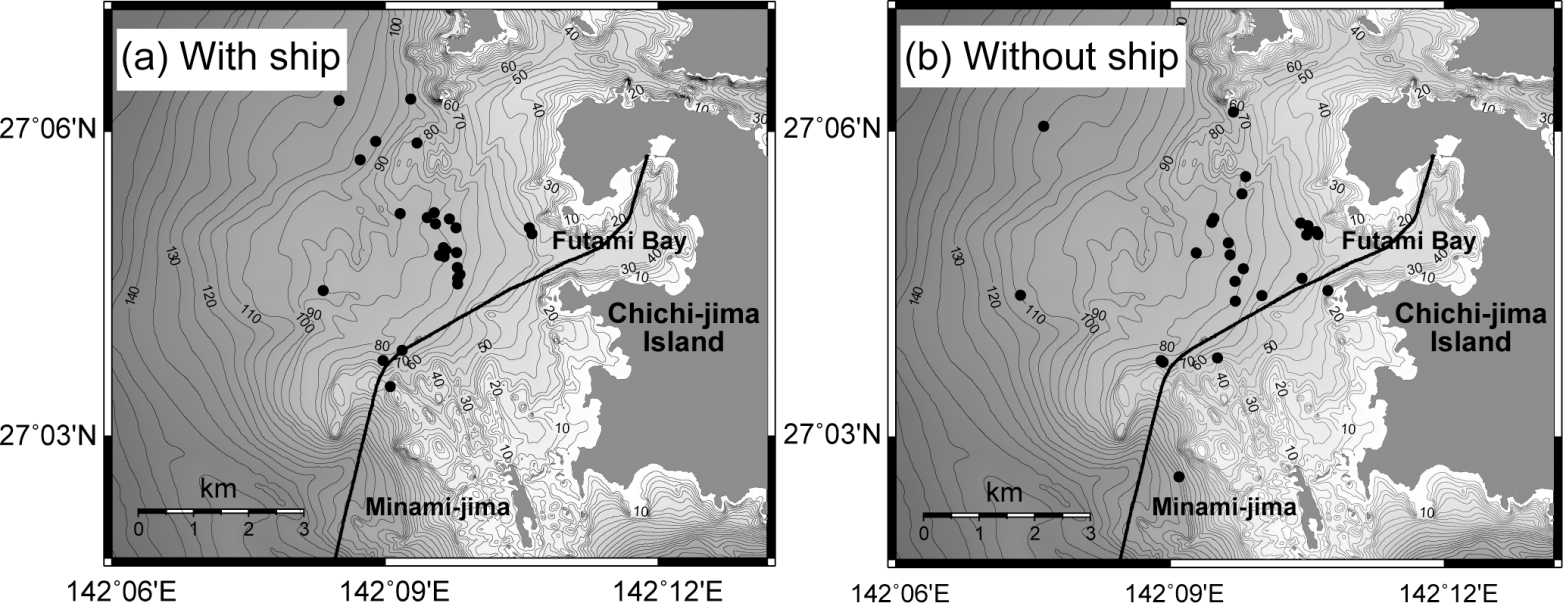
Estimated location of singers from 7:30 to 8:00 am. Black circles show each singer’s location calculated acoustically in 35 analyzed days. The ship trajectory (black line) on February 20 was drawn in both maps with or without the ship operating.

**Fig 5:**
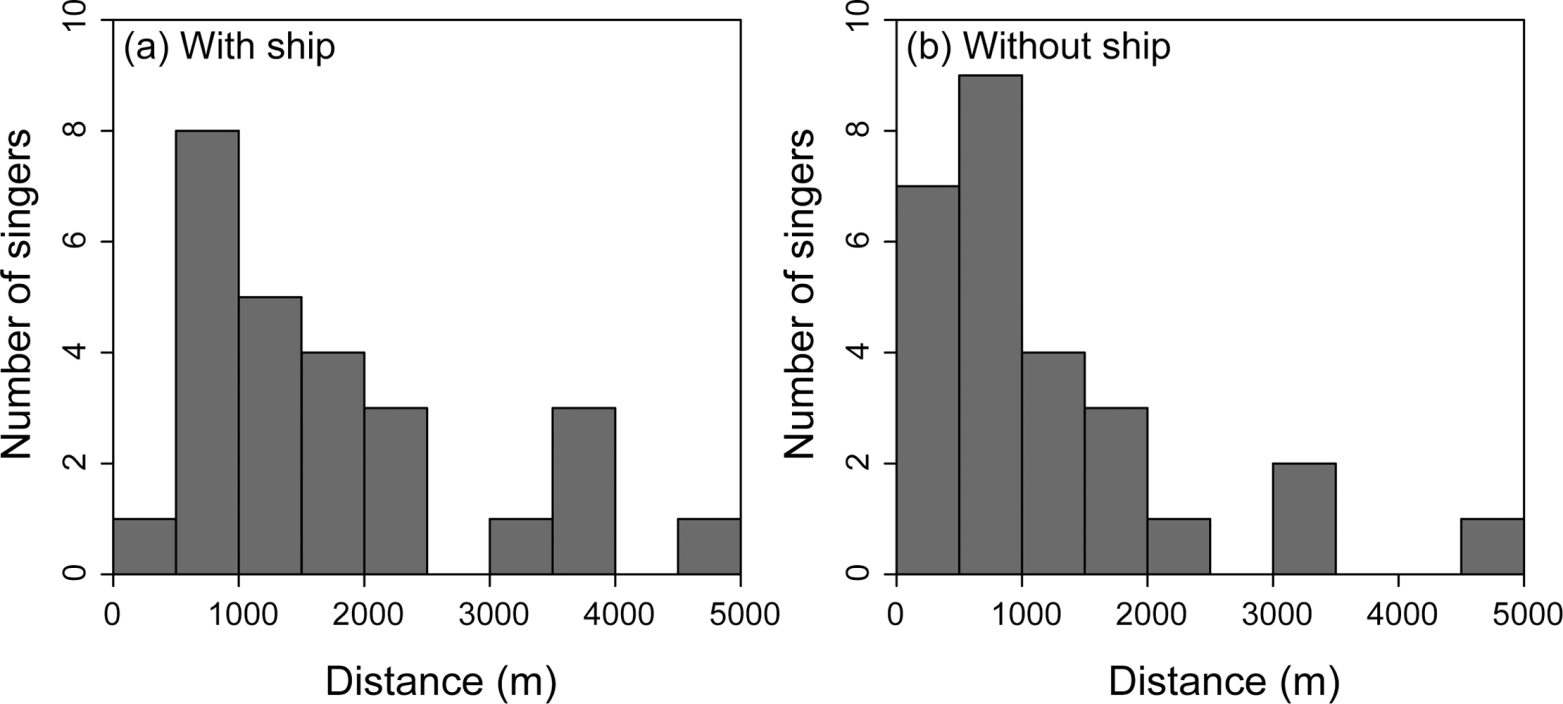
The nearest distance between the singers and the ship trajectory. The estimated location of each singer from 7:30 to 8:00 am that were used in the calculation. The mean of distance (a) with the ship was 1758 m, and (b) without the ship was 1210 m. The number of singing whales observed in each distance bin gradually reduced. Only one singer was found within 500 m from the ship trajectory on the day with the ship operating, wheras 7 singers were found on the day with no ship operating.

Accumulated distributions of singers during days with and without the ship were compared (Fig 4). Additionally, the closest distance from a singer to the ship trajectory was calculated. Distribution of the detected number of singers in each distance bin is depicted in Fig 5. There was a significant difference in the distribution of singers with and without the presence of the ship (Kolmogorov–Smirnov test, *P* = 0.028, Fig 5A). Obviously, a small number of singers were detected within 500 m of the ship trajectory when the ship was operating. When the analysis was confined to distances over 500 m, distribution of the number of singers related to the distance from the ship trajectory did not show a significant difference (Kolmogorov–Smirnov test, *P* = 0.348, Fig 5B).

**Fig 6:**
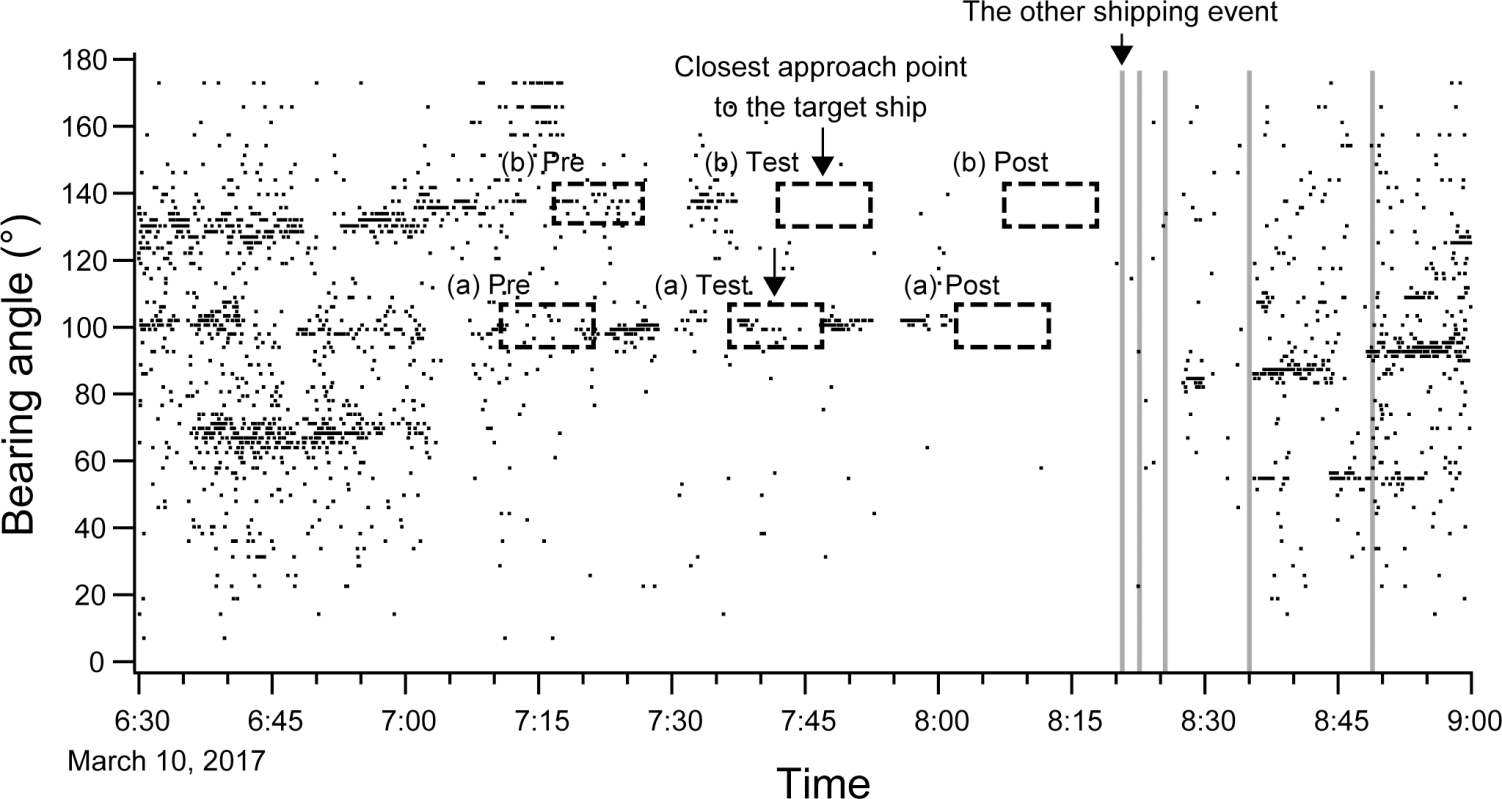
The bearing angle plots of all observed song units from 6:30 to 9:00 am on March 10, 2017. The dots show the detected units. Two singers could be recognised. Dashed boxes show each experiment period (pre, test, and post) and the down arrows indicate the closest approach point of the ship to the singer. The reactions of each singer differed. (a) The whale stopped singing after the ship passed by the test area. (b) The whale stopped singing before the ship passed by the test area. The minimum distance from singers to the ships were (a) 1180 m and (b) 235 m. Noises of other ships were also observed after 8:20 am (vertical gray line).

The bearing angle plots of all observed song units during pre-test, test, and post-test periods often showed a reduction or even termination of a song after the ship passed by (Fig 6). In this example, a clear sequence of song units could be observed during the pre-exposure period and continued during the test period, too (Fig 6(A)). However, the received number of song units decreased after the test period.

**Fig 7:**
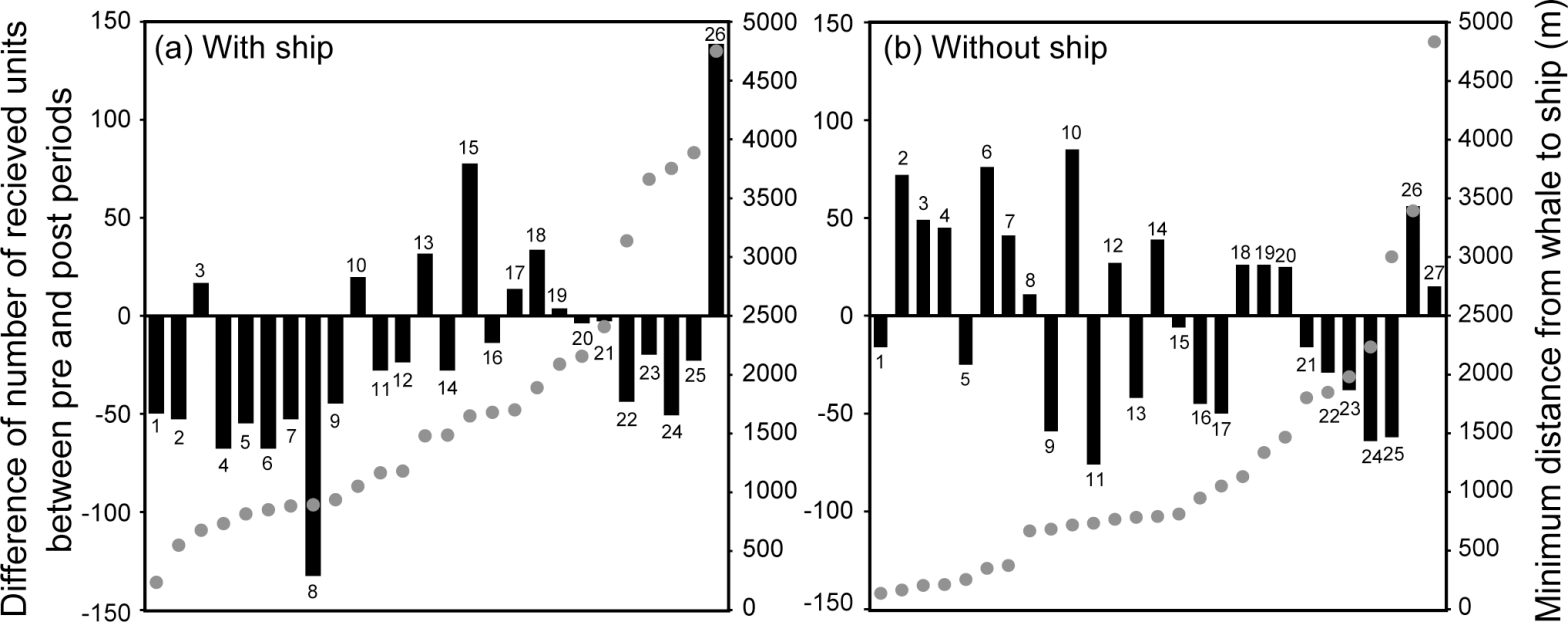
Relationship between singer’s location and an increase or decrease of song. These figures show the difference in the number of received units between pre- and post-test periods (black bars) and minimum distance from each whales to the target ship (gray circles) when the ship was (a) present and (b) absent. Without the ship operating, we calculated the distance between the singer and the virtual ship trajectory. When the ship was present, at distances up to 1200 m, 10 of 12 whales reduced song units. The number of received units after the ship passed was significantly less than that before the ship passed (Wilcoxon signed rank test, *P* = 0.005, n = 12). However, nearly same number of whales increased or decreased the unit production over the 1400 m range (Wilcoxon signed rank test, *P* = 0.975, n = 14). When the ship was absent, no trend could be observed under the 1200 m range from the ship trajectory (*P* = 0.528, n = 18). Observed whales are ordered from left to right acording to the minimum distance from the target ship (X-axis). Numbers in the graph show the serial number of observed whales.

The change in the received number of units according to the distance between singers and the ship trajectory was examined. Up to 1200 m, 10 of 12 whales reduced or terminated song, whereas nearly the same number of whales increased or decreased the unit production over the 1400 m range. Using a Wilcoxon signed rank test, it was shown that sound production after the ship passed was significantly less than that before the ship passed (*P* = 0.005, n = 12, Fig 7A, Table 2). On the other hand, no significant trend of increment or decline of the number of received units was observed when the distance to the ship trajectory was larger than 1400 m (*P* = 0.975, n = 14). In addition, no trend could be observed under the 1200 m range from the ship trajectory when no ship operated (*P* = 0.528, n = 18, Fig 7B).

**Table 2:** The results of comparison of song parameters between pre- and post-test periods.

Parameters	*P*-value (n)					
	With ship			Without ship		
	< 1200 m	> 1200 m	All singers	< 1200 m	> 1200 m	All singers
**Number of received units**	< 0.05 *	0.975	< 0.05 *	0.528	0.374	0.857
	(n = 12)	(n = 14)	(n = 26)	(n = 18)	(n = 9)	(n = 27)
**Minimum frequency**	0.401	0.925	0.615	0.407	0.110	0.112
	(n = 8)	(n = 14)	(n = 22)	(n = 17)	(n = 9)	(n = 26)
**Maximum frequency**	0.329	0.594	0.858	0.523	0.139	0.182
	(n = 8)	(n = 14)	(n = 22)	(n = 17)	(n = 9)	(n = 26)
**Sound pressure level**	0.208	0.109	0.426	0.210	0.214	0.073
	(n = 8)	(n = 14)	(n = 22)	(n = 17)	(n = 9)	(n = 26)
**Duration**	0.263	0.730	0.338	0.868	0.859	0.929
	(n = 8)	(n = 14)	(n = 22)	(n = 17)	(n = 9)	(n = 26)

*P*-value and the number of samples (n) used in a Wilcoxon signed rank test are shown in this table. Asterisk indicates that there is a significant difference between pre- and post-test periods.

Other parameters such as minimum and maximum frequency, duration, and received sound pressure level did not show any upward or downward trend between pre- and post- periods even when the dataset was restricted to a distance less than 1200 m (all *P* > 0.05, S1 Fig, Table 2).

As for the duration of noise effect, 9 of 12 whales that stopped singing clearly did not restart singing for at least 30 minutes after the target ship passed by if each singer remained at the same place, as shown in Fig 6. In these situations, shipping noises other than the target ship were also observed after 8:20 am, except in one case.

## Discussion

Shipping noise altered part of the humpback whales’ singing behavior. The effective distance was estimated to be between 500 m and 1200 m. A humpback whale whose distance to the target ship was 235 m stopped singing when the ship was approaching, and 9 whales whose distance was 500–1200 m reduced sound production or stopped singing after the ship passed by. However, no frequency or sound level modulation was observed in the presence of the ship. Hence, the main reaction of humpback whales was to stop singing either when the ship approached or after it passed by. In the present study, no other noise sources existed during the data collection. Manual exclusion of a small boat that passed by the experiment site removed any bias such that the observations were resticted to the effect of the specific (focal) shipping noise on individual humpback whales. The source level of the focal ship was 157 dB at 54 Hz, which is comparable to or lower than that of the song. The prominent frequency of the ship noise is lower than the fundamental vocal frequency range of the humpback whales in the Ogasawara Islands (100–800 Hz). At a distance of 500 m to 1200 m, propagation loss of the sound ranged from 54 dB to 62 dB if sound propagates spherically. The exposure level to a whale was 103 dB or 95 dB, which is even smaller than the received level of units analyzed in the present study (110–136 dB rms re 1 μPa). The speed of the subject ship was 7.7 m/s, meaning the ship travelled 1 km in 130 seconds. From the whales’ perspective, they can wait for 3 minutes for the noise level to revert to close to the background sound levels. The reaction of the singing behavior to the ship noise in the present study was quite limited, it was within several hundred meters. In a previous study, Risch et al. [44] suggested that the distance of low frequency noise effects on whale song could be up to 200 km. Noise received level ranged from 88–110 dB rms re 1 μPa, which was 5–22 dB above ambient noise levels in Risch et al. [44]. Yet, this may only occur in very calm waters. However, the difference in the background noise level and source level of noises narrowed the affected area in the Ogasawara waters.

We should note that the reduction of the song unit was more prominent when the ship completely disappeared from the observation area. Over the course of 15 minutes, the ship moved about 8.5–10 km. The noise level at the focal whale was then equivalent to the natural background noise level or even lower. Due to the limited number of focal whales, the period for which the noise effect lasted could not be determined. Moreover, to estimate the time range of the effects accurately, a cooling interval should be shifted. In our results, 9 of 12 whales stopped singing and did not restart singing until at least 30 minutes after the target ship had passed by the test area. Nevertheless, given that the operation of other ships (e.g., small boats and whale-watching boats) started to increase 30 minutes after the target ship passed by the test area, it was difficult to estimate how long the effect of the target ship lasted. However, it seemed that the effect of the target ship on the singing activity of humpback whales was temporary.

Acoustic observation can only detect phonating animals. Our data showed that, when the ship was operating, a singer did not occur on the ship trajectory, but this does not mean absence of humpback whales. Some of the whales may have terminated singing, while others could have been females or calves. Therefore, our conclusion is only applicable to singing males. Coverage area by the current passive acoustic system could be limited. For example, many singers were detected in west side of the monitoring array and few were detected from south area. As shown in Fig 1, the bathymetry around Minami-jima, which is in the south part of study area, is complicated and includes very shallow areas that could prevent sound propagation to the recorders even if there were singers in this area.

In the present study, the observed reactions of humpback whales were for them to cease singing or reduce the number of song units over at least 30 minutes when the ship was passing or after it had passed by. The acoustical response of marine mammals to anthropogenic noise varies [5]. Changing in call rate, and the modulating of the frequency, amplitude and duration of their vocalization have been reported (e.g., [18, 19, 45–47]). In baleen whales, blue whales (*Balaenoptera musculus*) increased their call rate during seismic exploration [45], and fin whales (*Balaenoptera physalus*) produced 20 Hz calls with shorter duration and lower frequency than usual when exposed to a seismic airgun noise [18]. North Atlantic right whales (*Eubalaena glacialis*) increased the amplitude of their calls with increasing background noise level [46]. As for the responses of humpback whales to shipping noise, decrease in singing activity and shortening of song duration were observed [14, 35]. From these reviews, it is possible that humpback whales have a tendency to stop or decrease their song when the noise source level is higher. Decrease in singers or songs nearby a ship trajectory observed in our study concurs with these findings. Remarkably, behavioral changes were observed with a ship’s passing except for when a whale was near a shipping line (<500 m). This result indicates that whales which were under a ship noise exposure continued to sing as usual. The source level of the target ship noise was 157 dB re 1μPa, and the received level at each whales’ position (500–1200 m from shipping line) was at most 8 dB higher than or almost the same as the background noise level (95 dB). In addition, the source level range of a whale’s song, which is estimated to be from 151 dB to 173 dB rms re 1μPa [29], is much higher than the received level of the ship noise. Hence, they may not have needed to modulate their song parameters to avoid the masking effect by shipping noise. Then, in terms of cost to the individual, whales might choose to cease vocalization and move away from noise source rather than adjusting their calls under a high noise level condition, as a whale close to the shipping lane stopped singing. In the present case, it is suggested that the effect of noise generated by the focal passenger-cargo liner is limited and the recovery of whales is fast in the Ogasawara waters.

The reason why the reaction occurred after a shipping event may have been related to the directivity of the ship noise and the threshold of the sound exposure level received by each individual. As the highest noise level emanated from the rear of the target ship, whales just behind the ship would have been the most strongly influenced by noise. In addition, given that ship noise raises stress levels in right whales, and it was considered to be applicable to all baleen whales [10], stresses imposed by the continuous exposure to noise might elicit changes in singing behavior after a ship’s passage. For future research, we need to examine the relationships between sound exposure level and the reaction of whales.

## Supporting information

S1 File The consent form provided by Ogasawara Fisheries Cooperative Association (written in Japanese). (PDF)

S1 Appendix A long baseline hydrophone array using two static recorders to monitor the singing behavior of humpback whales. (DOCX)

S1 Fig The variation between pre- and post-test periods in: (a) the mean of minimum frequency (Hz) with ship and (b) without ship; (c) the mean of maximum frequency (Hz) with ship and (d) without ship; (e) the mean of received sound level (dB rms re 1μPa) with ship and (f) without ship; (g) the mean of duration of units with ship and (h) without ship. The gray circles show the minimum distance from each whales to the target ship.
(TIF)

S1 Table The number of received units during pre-test, test and post-test periods. (DOCX)

S2 Table Mean (± SD) of minimum frequency (Hz) of received units during pre-test, test and post-test periods. (DOCX)

S3 Table Mean (± SD) of maximum frequency (Hz) of received units during pre-test, test and post-test periods. (DOCX)

S4 Table Mean (± SD) of sound pressure level (dB rms re 1μPa) of received units during pre-test, test and post-test periods. (DOCX)

S5 Table Mean (± SD) of duration (sec) of received units during pre-test, test and post-test periods. (DOCX)
