# Supplementary material for: Change in singing behavior of humpback whales caused by shipping noise
Source: PLoS One. 2018 Oct 24;13(10):e0204112. doi: 10.1371/journal.pone.0204112 (PMC6200181; doi:10.1371/journal.pone.0204112)
Supplement: S1 File — (PDF) [file pone.0204112.s001.pdf]

同　　意　　書

平成 28 年 12 月 12 日

小笠原ホエールウオッチング協会
代表理事　　吉　井　信　秋　殿

東京都小笠原村父島字奥村
小笠原島漁業協同組合
代表理事組合長　菊池勝貴

小笠原におけるクジラ類の鳴音および行動調査に関する同意について（回答）

平成 28 年 12 月 8 日付けで依頼のあった、水中マイク設置について、漁業の操業及び船舶への航行に支障のないことと、調査終了後の撤去を条件として同意いたします。

同　　意　　書

平成 29 年 3 月 7 日

小笠原ホエールウオッチング協会
代表理事　　吉　井　信　秋　殿

東京都小笠原村父島字奥村
小笠原島漁業協同組合
代表理事組合長　菊池勝貴

小笠原におけるクジラ類の発声行動調査及びクジラ類の鳴音および行動調査の期間延長の同意について（回答）

平成 28 年 12 月 12 日付けで同意した調査の期間延長について同意致します。

漁業の操業及び船舶への航行に支障のないことと、調査終了後のすみやかな撤去を条件として同意いたします。

(In English)

Letter of consent

December 12, 2016

Mr. Nobuaki Yoshii
Representative Director
Ogasawara Whale Watching Association

Katsutaka Kikuchi
Representative Director and Union President
Ogasawara Fisheries Cooperative Association
Aza-Okumura, Chichijima, Ogasawara-mura, Tokyo

Response to your request for the consent to the research of calling behavior of whales in the Ogasawara waters

Dear Mr. Nobuaki Yoshii,

I agree to the deployment of underwater recorders in the Ogasawara waters which was requested on December 8, 2016. I agree to that on condition that the recorders do not prevent the operation of fishing and ship navigation and are recovered after the end of the research.

Sincerely yours,

(In English)

Letter of consent

March 7, 2017

Mr. Nobuaki Yoshii
Representative Director
Ogasawara Whale Watching Association

Katsutaka Kikuchi
Representative Director and Union President
Ogasawara Fisheries Cooperative Association
Aza-Okumura, Chichijima, Ogasawara-mura, Tokyo

Response to your request for the consent to the extension of the research period of calling behavior of whales in the Ogasawara waters

Dear Mr. Nobuaki Yoshii,

I agree to the extension of the period of your research which I agreed on December 12, 2016. I agree to that on condition that the recorders do not prevent the operation of fishing and ship navigation and are recovered after the end of the research.

Sincerely yours,
