# Supplementary material for: Change in singing behavior of humpback whales caused by shipping noise
Source: PLoS One. 2018 Oct 24;13(10):e0204112. doi: 10.1371/journal.pone.0204112 (PMC6200181; doi:10.1371/journal.pone.0204112)
Supplement: S1 Appendix — (DOCX) [file pone.0204112.s002.docx]

**S1 Appendix:**

**A long baseline hydrophone array using two static recorders to monitor the singing behavior of humpback whales**

# 1. Detail methods

## **1.1 Recording systems**

Two autonomous stereo recording systems (AUSOMS-mini stereo; Aquasound Inc., Kyoto, Japan) were deployed, 3.0 km apart, off Futami Bay, Chichi-jima, Ogasawara, Japan. The water depth at the recording station was 40 m and the recorder was suspended horizontally at 20 m, in the middle of the water column. The top buoy was located at a depth of 4 m to help avoid possible collisions with small boats (Fig S1). For retrieval, we located the recorder using GPS and confirmed the position visually from the ocean surface. Permits and approvals for the deployment of recorders in the Ogasawara waters were obtained from Ogasawara Fisheries Cooperative Association.

Because of the large distance between the two recorders (3.0 km), the time difference in sound arrival at the two recorders was approximately ± 2.0 s. To synchronize the clocks of the recorders, a sequence of pulse sounds was recorded simultaneously. At the beginning and the end of the recording period, the two autonomous recorders were placed in a water-filled bucket which was hit several times. This primitive and simple method to synchronize the clocks of the recorders was sufficient to separate out individual song units using the long baseline array. The clocks of two recorders could be synchronized within 10 ms, which is small enough in comparison with the maximum time difference of 2.0 s of the observation array.

Because we intended to perform continuous long-term recording, a compressed data format (mp3, 128 kbps) was used to reduce memory consumption. This compressed format allowed recording at a frequency of up to 17 kHz, which is well above the fundamental frequency of humpback whale songs (below 1 kHz). Since the mp3 format is compressed, the original waveform or phase information may be lost. In the proposed protocol, we used contour shape correlations to measure the time difference in the arrival of each unit of song, as these were less affected by the recording format compared with the waveform cross-correlation between the two recordings.





**Fig S1.** **The deployment system of a stereo recorder.**

The recorder was horizontally suspended at a depth of 20 m using buoys, with a marker buoy situated at 4 m below the water surface. This recording system was deployed at the north and south stations, off the coast of Chichi-jima.

## **1.2 Time synchronization protocols**

The absolute times at which the pulse sounds were recorded at the beginning and end of the recording period, at the north and south recording stations, were denoted as TNb, TNe, TSb, and TSe, respectively. By using a waveform viewer, such as Adobe Audition (Adobe Systems Inc., USA) or Audacity (http://www.audacityteam.org/), these absolute times can be measured easily. Note that the timestamp of the sound file is the absolute time at the end of each recording. The initial time shift of different recorders (B) is given by:

B = TNb - TSb (Eq. 1)

This initial offset is due mostly by the resolution of the internal clocks of the recorders, which is generally 1 s. In addition to the initial offset, drift of the crystal clock causes a gradual shift in the recorded time. The difference in time drift between the two recorders (A) is given by:

A = [(TNb-TSb)-(TNe-TSe)] / (TNe-TNb) (Eq. 2)

The time difference between the two recorders (X) at arbitrary time (T) is given by:

X = AT + B (Eq. 3)

Passive acoustic recording may continue over a long duration, of weeks or even months, such that many large sound files are created. In our case, sixty 8-h sound files, which corresponded to 20 days of recording, were obtained from a single session of passive acoustic recording. All of the sound files of the two monitoring stations were divided into short (10 min) sections. Pairs of files were synchronized using a MATLAB-based synchronization program. The program can combine monaural recordings taken at the two stations into a stereo audio file; 10-min sections recorded at channel 2 were shifted using Eq. (3) depending on the time elapsed, T, from the start of the observation period. The time drift seen for each 10-min file is negligible compared with the difference in the time of arrival of the sounds at the two stations.

## **1.3 Data processing to calculate the time difference in sound arrival between the two recording stations**

Once the files had been time-synchronized, the time difference between each contour of a song unit recorded at the two stations could be calculated. For this calculation, waveform cross-correlation, or image matching of song unit contours in the time-frequency domain, can be used. We used spectrogram correlation [1] rather than direct waveform cross-correlation. Cross-correlation is strongly affected by background noise and reverberations, which prevents measurement of the true time difference in the arrival of each sound source at the recording stations, especially in a situation where multiple whales are phonating simultaneously. In the present study area, two or more whales produced sounds in different locations during the entire day. Instead of using a “naïve” cross correlation, four-step image matching of the contour shapes of song sequences was done.

First, the spectrogram of the channel 1 recording was calculated. Then, a denoising procedure was applied to the spectrogram. The locations of all prominent peaks of the power spectrum, from 100 to 2,000 Hz for each size of fast Fourier transform (FFT) (2^12^=4096 for a 44.1 kHz sampling frequency, 92.8 ms duration, blackman window), were extracted as a vector. Neighboring sections overlapped by 50% in the time domain. Consequently, the time resolution of the FFT was 46.4 ms. Here, “prominent” means that the local maximum peaks in the frequency domain of the power spectrum have preset elevations of spectrum intensity compared with those at neighboring local minima (for more details, refer to the “findpeaks” command of MATLAB). In the MATLAB code, the “MinPeakProminence” and “MinPeakHeight” were set to 20 dB and −40 dB, respectively. A series of prominent vectors obtained at 46.4 ms intervals was stored as a matrix for up to 1 min. The matrix represented the most prominent spectrum peaks in the time and frequency domains. During the denoising procedure, all peaks in the matrix without association of other prominent peaks occurring within 53.8 Hz (five times the frequency resolution of the current FFT) in neighboring areas of the time bins were deleted, because it was considered as an isolated local peak of the power spectrum and did not form a continuous contour.

Second, individual contours were identified by connecting prominent peaks within the matrix (Fig S2, left), by starting with a peak in the power spectrum and connecting it to the closest peak (within ± 53.8 Hz) in the next time bin. If no other peak could be found within this frequency range, it was deemed the end of a contour. The identification number of each contour was stored in a different matrix of the same size as that of the original matrix of prominent peaks.

Third, a matrix of prominent peaks recorded at channel 2 was produced in the same manner. The elements comprising the prominent peaks were all set to 1 to avoid oversensitive detection when using image cross correlation. The denoising procedure was also applied but the contour connection method was not used (Fig S2, right).

Finally, image cross correlation was done for each spectrogram contour. The prominent peak matrix of the recording at the north station, designated by the contour matrix (thick rectangle in Fig S2, left), was used as a template. The template prominent peak matrix was shifted horizontally in the time domain and correlated with the prominent peak matrix of the recording at the south station (Fig S2, right). Contours including ≥ 5 prominent peaks were correlated to eliminate very short sounds. The template prominent peak matrix recorded at the north station was shifted by ± 3 s compared with the matrix recorded at channel 2. The ± 3 s shift included a 50% additional margin to the physical maximum time difference. A frequency shift in the minimum resolution of up to 53.8 Hz was allowed. The time shift in the maximum cross correlation value for each spectrogram contour was stored.



**Fig S2. A connected contour matrix with identification numbers of song units recorded at the north station (left) was produced.** Each spectrogram contour in the matrix was used as a template and correlated with the locations of the prominent peaks recorded at the south station (right). The time difference in arrival of a sound at each recording station could be measured separately for each spectrogram contour. The time shifts in the upper and lower contours are indicated by the gray arrows.

# 2. Results of song detection

The time differences for detected spectrogram contours during 9 h period are shown in Fig S3. Thick black lines and scattered noise can be seen. One black line of the time differences of sound arrival corresponds to a change in direction of a singing whale. Fig S3 shows that the whales moved or drifted gradually, corresponding to changes in the relative bearing angle on the monitoring array. In many cases, the song sequences continued for 3 h or more. It is also clear that multiple sound sources existed simultaneously at different bearing angles. Fig S3 illustrates the minimum number of singing whales at a given time. The beginning and end of a trace could correspond to the times at which a whale stopped singing, swam out of the observable range, or reduced its source level. The magnified area within the rectangle in Fig S3 shows that each trace was characterized by periodic breaks (Fig S3, right inset). Song sequences restarted again, emanating from a similar direction, after several minutes of silence; this occurred three to four times per hour.





**Fig S3. Example of the time difference in arrival of a sound at the two recording stations, observed using the long baseline array.** Different sound sources (i.e., individual whales) could be distinguished. Two to five whales produced songs simultaneously during the 9-h recording period. Singing by multiple whales was observed frequently over the entire day. The magnified image (right) shows periodic breaks in a sequence of song units, indicated by black arrows, which may have corresponded to the respiration interval of each whale.

**References**

1. Tiemann CO and Porter MB. Localization of marine mammals near Hawaii using an acoustic propagation model. J Acoust Soc Am. 2004; 115: 2834–2843.

The English in this document has been checked by at least two professional editors, both native speakers of English. For a certificate, please see:

http://www.textcheck.com/certificate/4kGbvq
