# Supplementary figures and images for: Change in singing behavior of humpback whales caused by shipping noise
Source: PLoS One. 2018 Oct 24;13(10):e0204112. doi: 10.1371/journal.pone.0204112 (PMC6200181; doi:10.1371/journal.pone.0204112)

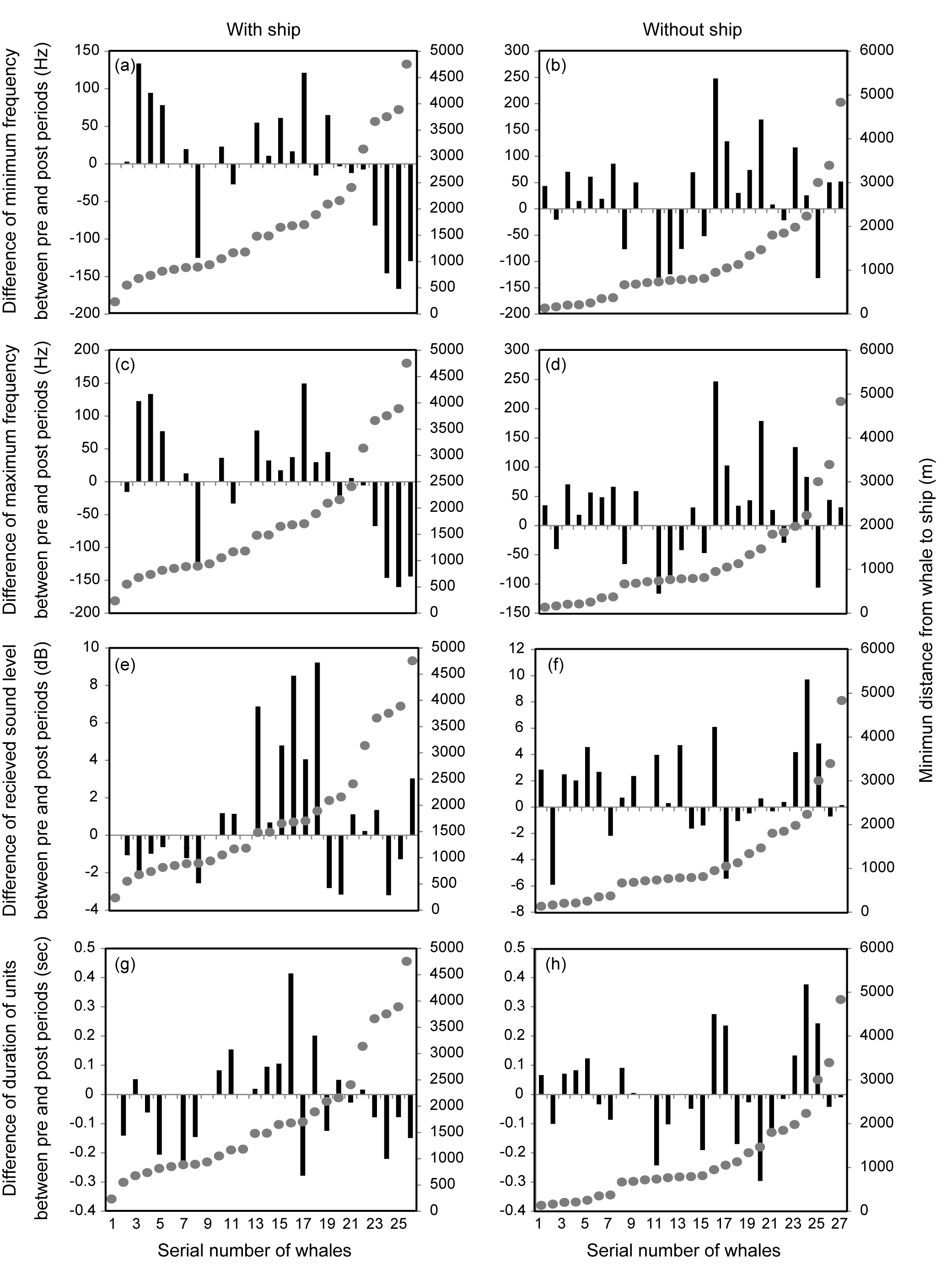

Supplement: S1 Fig — The variation between pre- and post-test periods in: (a) the mean of minimum frequency (Hz) with ship and (b) without ship; (c) the mean of maximum frequency (Hz) with ship and (d) without ship; (e) the mean of received sound level (dB rms re 1μPa) with ship and (f) without ship; (g) the mean of duration of units with ship and (h) without ship. The gray circles show the minimum distance from each whales to the target ship. (TIF) [file pone.0204112.s003.tif]
