# Supplementary material for: Change in singing behavior of humpback whales caused by shipping noise
Source: PLoS One. 2018 Oct 24;13(10):e0204112. doi: 10.1371/journal.pone.0204112 (PMC6200181; doi:10.1371/journal.pone.0204112)
Supplement: S1 Table — (DOCX) [file pone.0204112.s004.docx]

**S1 Table. The number of received units during pre-test, test and post-test periods.**

| **With ship** | | | | **Without ship** | | | |
| --- | --- | --- | --- | --- | --- | --- | --- |
| **Distance (m)** | **Pre** | **Test** | **Post** | **Distance (m)** | **Pre** | **Test** | **Post** |
| 235 | 49 | 0 | 0 | 137 | 70 | 110 | 86 |
| 551 | 108 | 78 | 56 | 164 | 103 | 110 | 31 |
| 677 | 84 | 85 | 100 | 203 | 147 | 153 | 98 |
| 734 | 81 | 50 | 14 | 211 | 164 | 32 | 119 |
| 816 | 107 | 60 | 53 | 254 | 42 | 46 | 67 |
| 851 | 67 | 160 | 0 | 350 | 146 | 111 | 70 |
| 885 | 62 | 116 | 10 | 374 | 82 | 99 | 41 |
| 894 | 143 | 153 | 11 | 668 | 125 | 61 | 114 |
| 937 | 44 | 2 | 0 | 682 | 26 | 98 | 85 |
| 1052 | 44 | 43 | 63 | 718 | 85 | 61 | 0 |
| 1166 | 59 | 43 | 32 | 734 | 3 | 95 | 79 |
| 1180 | 23 | 34 | 0 | 767 | 102 | 52 | 75 |
| 1480 | 32 | 74 | 63 | 784 | 6 | 116 | 48 |
| 1487 | 80 | 62 | 53 | 792 | 104 | 46 | 65 |
| 1650 | 2 | 28 | 79 | 812 | 68 | 39 | 74 |
| 1681 | 142 | 146 | 129 | 948 | 2 | 70 | 47 |
| 1701 | 34 | 67 | 47 | 1051 | 13 | 63 | 63 |
| 1890 | 26 | 7 | 59 | 1130 | 98 | 43 | 72 |
| 2090 | 60 | 74 | 63 | 1335 | 56 | 46 | 30 |
| 2157 | 91 | 71 | 88 | 1466 | 91 | 117 | 66 |
| 2409 | 91 | 81 | 89 | 1802 | 26 | 56 | 42 |
| 3138 | 74 | 51 | 31 | 1848 | 122 | 111 | 151 |
| 3663 | 42 | 16 | 23 | 1981 | 83 | 44 | 121 |
| 3754 | 100 | 71 | 50 | 2233 | 5 | 18 | 69 |
| 3888 | 119 | 170 | 97 | 3002 | 21 | 40 | 83 |
| 4752 | 2 | 56 | 140 | 3393 | 191 | 128 | 135 |
|  |  |  |  | 4833 | 49 | 85 | 34 |
