# Supplementary material for: Change in singing behavior of humpback whales caused by shipping noise
Source: PLoS One. 2018 Oct 24;13(10):e0204112. doi: 10.1371/journal.pone.0204112 (PMC6200181; doi:10.1371/journal.pone.0204112)
Supplement: S2 Table — (DOCX) [file pone.0204112.s005.docx]

**S2 Table. Mean (± SD) of minimum frequency (Hz) of received units during pre-test, test and post-test periods.**

| **With ship** | | | | **Without ship** | | | |
| --- | --- | --- | --- | --- | --- | --- | --- |
| **Distance (m)** | **Pre** | **Test** | **Post** | **Distance (m)** | **Pre** | **Test** | **Post** |
| 235 | 362 (198) | NA | NA | 137 | 413 (193) | 457 (222) | 457 (191) |
| 551 | 368 (298) | 295 (161) | 371 (180) | 164 | 390 (249) | 318 (156) | 369 (138) |
| 677 | 223 (90) | 327 (146) | 356 (222) | 203 | 349 (225) | 411 (308) | 419 (233) |
| 734 | 429 (284) | 425 (163) | 524 (139) | 211 | 355 (185) | 362 (163) | 370 (175) |
| 816 | 337 (165) | 346 (187) | 415 (204) | 254 | 351 (162) | 368 (153) | 412 (202) |
| 851 | 386 (304) | 360 (215) | NA | 350 | 424 (248) | 352 (242) | 443 (324) |
| 885 | 307 (162) | 386 (170) | 326 (246) | 374 | 313 (193) | 374 (235) | 399 (132) |
| 894 | 438 (298) | 329 (172) | 313 (153) | 668 | 395 (238) | 444 (284) | 318 (140) |
| 937 | 396 (171) | 202 (99) | NA | 682 | 339 (181) | 342 (210) | 389 (204) |
| 1052 | 353 (176) | 407 (191) | 376 (195) | 718 | 441 (369) | 371 (263) | NA |
| 1166 | 397 (143) | 446 (102) | 370 (121) | 734 | 473 (6) | 336 (162) | 327 (137) |
| 1180 | 423 (273) | 462 (83) | NA | 767 | 554 (402) | 437 (259) | 430 (243) |
| 1480 | 324 (179) | 438 (239) | 379 (222) | 784 | 520 (239) | 412 (231) | 444 (199) |
| 1487 | 378 (206) | 357 (216) | 389 (160) | 792 | 379 (161) | 371 (244) | 449 (293) |
| 1650 | 407 (251) | 336 (134) | 468 (315) | 812 | 478 (223) | 370 (161) | 426 (201) |
| 1681 | 397 (227) | 398 (215) | 414 (250) | 948 | 154 (15) | 302 (132) | 402 (164) |
| 1701 | 392 (122) | 448 (295) | 513 (265) | 1051 | 227 (107) | 314 (197) | 355 (152) |
| 1890 | 335 (97) | 337 (149) | 320 (171) | 1130 | 313 (152) | 304 (133) | 343 (183) |
| 2090 | 523 (333) | 335 (156) | 588 (370) | 1335 | 373 (188) | 361 (133) | 447 (152) |
| 2157 | 392 (175) | 393 (179) | 389 (183) | 1466 | 341 (150) | 412 (216) | 511 (190) |
| 2409 | 400 (252) | 381 (222) | 388 (225) | 1802 | 459 (232) | 419 (154) | 467 (218) |
| 3138 | 382 (158) | 476 (218) | 375 (151) | 1848 | 410 (241) | 362 (196) | 388 (239) |
| 3663 | 416 (133) | 408 (286) | 335 (228) | 1981 | 262 (134) | 604 (157) | 379 (238) |
| 3754 | 502 (396) | 371 (210) | 357 (157) | 2233 | 399 (93) | 337 (41) | 425 (144) |
| 3888 | 526 (379) | 508 (306) | 360 (212) | 3002 | 378 (185) | 313 (162) | 247 (110) |
| 4752 | 455 (0) | 459 (213) | 326 (167) | 3393 | 430 (288) | 406 (328) | 481 (343) |
|  |  |  |  | 4833 | 318 (143) | 371 (200) | 370 (236) |
