# Supplementary material for: Change in singing behavior of humpback whales caused by shipping noise
Source: PLoS One. 2018 Oct 24;13(10):e0204112. doi: 10.1371/journal.pone.0204112 (PMC6200181; doi:10.1371/journal.pone.0204112)
Supplement: S3 Table — (DOCX) [file pone.0204112.s006.docx]

**S3 Table. Mean (± SD) of maximum frequency (Hz) of received units during pre-test, test and post-test periods.**

| **With ship** | | | | **Without ship** | | | |
| --- | --- | --- | --- | --- | --- | --- | --- |
| **Distance (m)** | **Pre** | **Test** | **Post** | **Distance (m)** | **Pre** | **Test** | **Post** |
| 235 | 389 (190) | NA | NA | 137 | 469 (196) | 508 (220) | 504 (184) |
| 551 | 428 (290) | 362 (158) | 412 (174) | 164 | 450 (247) | 380 (175) | 410 (128) |
| 677 | 273 (82) | 394 (154) | 395 (207) | 203 | 382 (221) | 477 (297) | 452 (222) |
| 734 | 518 (285) | 511 (174) | 651 (201) | 211 | 404 (186) | 395 (163) | 423 (180) |
| 816 | 402 (161) | 406 (186) | 479 (200) | 254 | 459 (146) | 458 (155) | 516 (193) |
| 851 | 439 (300) | 451 (192) | NA | 350 | 471 (240) | 387 (240) | 520 (321) |
| 885 | 391 (138) | 465 (160) | 404 (245) | 374 | 363 (187) | 434 (220) | 429 (129) |
| 894 | 507 (295) | 398 (167) | 379 (136) | 668 | 449 (233) | 509 (273) | 384 (147) |
| 937 | 475 (194) | 251 (152) | NA | 682 | 404 (159) | 398 (204) | 463 (194) |
| 1052 | 415 (171) | 478 (197) | 451 (188) | 718 | 511 (355) | 446 (255) | NA |
| 1166 | 445 (162) | 494 (133) | 412 (130) | 734 | 495 (12) | 385 (164) | 378 (130) |
| 1180 | 471 (276) | 547 (120) | NA | 767 | 608 (405) | 505 (264) | 514 (226) |
| 1480 | 366 (192) | 485 (221) | 444 (223) | 784 | 567 (237) | 492 (230) | 525 (190) |
| 1487 | 412 (215) | 403 (204) | 445 (160) | 792 | 453 (163) | 417 (238) | 484 (294) |
| 1650 | 504 (236) | 400 (124) | 521 (327) | 812 | 533 (216) | 426 (187) | 486 (208) |
| 1681 | 452 (218) | 453 (211) | 490 (240) | 948 | 224 (99) | 390 (146) | 470 (163) |
| 1701 | 433 (125) | 512 (295) | 583 (267) | 1051 | 316 (118) | 383 (181) | 419 (146) |
| 1890 | 356 (96) | 366 (166) | 386 (185) | 1130 | 378 (153) | 377 (131) | 411 (175) |
| 2090 | 590 (344) | 396 (152) | 635 (385) | 1335 | 460 (194) | 422 (150) | 504 (155) |
| 2157 | 454 (195) | 433 (190) | 433 (186) | 1466 | 390 (152) | 456 (219) | 569 (205) |
| 2409 | 448 (236) | 456 (223) | 454 (228) | 1802 | 504 (232) | 486 (174) | 531 (221) |
| 3138 | 424 (165) | 523 (208) | 418 (152) | 1848 | 474 (243) | 420 (201) | 445 (233) |
| 3663 | 448 (127) | 451 (268) | 381 (225) | 1981 | 306 (133) | 660 (163) | 440 (237) |
| 3754 | 557 (381) | 441 (205) | 411 (167) | 2233 | 414 (90) | 360 (46) | 497 (154) |
| 3888 | 611 (367) | 583 (297) | 451 (202) | 3002 | 430 (190) | 394 (154) | 324 (96) |
| 4752 | 536 (69) | 529 (215) | 392 (151) | 3393 | 504 (285) | 475 (337) | 548 (339) |
|  |  |  |  | 4833 | 377 (137) | 422 (196) | 408 (229) |
