# Supplementary material for: Change in singing behavior of humpback whales caused by shipping noise
Source: PLoS One. 2018 Oct 24;13(10):e0204112. doi: 10.1371/journal.pone.0204112 (PMC6200181; doi:10.1371/journal.pone.0204112)
Supplement: S4 Table — (DOCX) [file pone.0204112.s007.docx]

**S4 Table. Mean (± SD) of sound pressure level (dB rms re 1µPa) of received units during pre-test, test and post-test periods.**

| **With ship** | | | | **Without ship** | | | |
| --- | --- | --- | --- | --- | --- | --- | --- |
| **Distance (m)** | **Pre** | **Test** | **Post** | **Distance (m)** | **Pre** | **Test** | **Post** |
| 235 | 117 (3) | NA | NA | 137 | 117 (3) | 120 (5) | 119 (5) |
| 551 | 124 (6) | 123 (5) | 123 (6) | 164 | 123 (6) | 123 (5) | 117 (4) |
| 677 | 118 (3) | 118 (3) | 116 (3) | 203 | 120 (5) | 122 (6) | 123 (7) |
| 734 | 131 (6) | 128 (5) | 130 (3) | 211 | 119 (4) | 120 (5) | 121 (5) |
| 816 | 122 (4) | 121 (4) | 121 (4) | 254 | 126 (4) | 129 (4) | 130 (5) |
| 851 | 125 (7) | 125 (6) | NA | 350 | 121 (6) | 119 (4) | 123 (5) |
| 885 | 123 (6) | 123 (4) | 121 (6) | 374 | 120 (5) | 123 (6) | 118 (4) |
| 894 | 122 (5) | 122 (5) | 120 (5) | 668 | 123 (6) | 123 (6) | 123 (6) |
| 937 | 123 (5) | 121 (3) | NA | 682 | 120 (4) | 122 (6) | 122 (6) |
| 1052 | 119 (5) | 120 (4) | 120 (5) | 718 | 125 (7) | 126 (7) | NA |
| 1166 | 119 (4) | 118 (3) | 120 (4) | 734 | 114 (1) | 120 (5) | 118 (3) |
| 1180 | 119 (5) | 119 (4) | NA | 767 | 127 (6) | 124 (7) | 128 (6) |
| 1480 | 117 (3) | 119 (5) | 124 (5) | 784 | 124 (4) | 126 (6) | 129 (5) |
| 1487 | 118 (4) | 120 (5) | 119 (4) | 792 | 121 (6) | 117 (4) | 119 (5) |
| 1650 | 119 (1) | 122 (4) | 124 (5) | 812 | 123 (6) | 119 (4) | 122 (6) |
| 1681 | 122 (5) | 121 (6) | 130 (5) | 948 | 118 (2) | 124 (5) | 124 (6) |
| 1701 | 119 (4) | 122 (4) | 123 (5) | 1051 | 125 (5) | 121 (4) | 119 (4) |
| 1890 | 116 (2) | 122 (7) | 125 (7) | 1130 | 123 (5) | 120 (3) | 121 (4) |
| 2090 | 124 (5) | 122 (5) | 121 (4) | 1335 | 121 (4) | 119 (5) | 120 (5) |
| 2157 | 121 (5) | 119 (4) | 118 (4) | 1466 | 122 (5) | 122 (4) | 123 (5) |
| 2409 | 123 (5) | 127 (5) | 124 (5) | 1802 | 121 (4) | 121 (4) | 121 (4) |
| 3138 | 120 (4) | 121 (4) | 121 (5) | 1848 | 125 (7) | 124 (6) | 125 (7) |
| 3663 | 116 (3) | 118 (5) | 117 (3) | 1981 | 119 (3) | 121 (3) | 123 (6) |
| 3754 | 125 (7) | 125 (6) | 122 (5) | 2233 | 114 (2) | 115 (2) | 124 (5) |
| 3888 | 128 (6) | 126 (6) | 127 (5) | 3002 | 117 (4) | 122 (4) | 122 (4) |
| 4752 | 121 (1) | 124 (6) | 124 (6) | 3393 | 128 (8) | 129 (7) | 127 (7) |
|  |  |  |  | 4833 | 119 (5) | 121 (5) | 119 (5) |
