# Supplementary material for: Change in singing behavior of humpback whales caused by shipping noise
Source: PLoS One. 2018 Oct 24;13(10):e0204112. doi: 10.1371/journal.pone.0204112 (PMC6200181; doi:10.1371/journal.pone.0204112)
Supplement: S5 Table — (DOCX) [file pone.0204112.s008.docx]

**S5 Table. Mean (± SD) of duration (sec) of received units during pre-test, test and post-test periods.**

| **With ship** | | | | **Without ship** | | | |
| --- | --- | --- | --- | --- | --- | --- | --- |
| **Distance (m)** | **Pre** | **Test** | **Post** | **Distance (m)** | **Pre** | **Test** | **Post** |
| 235 | 0.53 (0.27) | NA | NA | 137 | 0.56 (0.29) | 0.58 (0.44) | 0.63 (0.42) |
| 551 | 0.69 (0.46) | 0.70 (0.43) | 0.55 (0.36) | 164 | 0.69 (0.50) | 0.74 (0.50) | 0.59 (0.40) |
| 677 | 0.47 (0.23) | 0.45 (0.23) | 0.52 (0.21) | 203 | 0.69 (0.42) | 0.58 (0.42) | 0.76 (0.45) |
| 734 | 0.80 (0.52) | 0.79 (0.56) | 0.74 (0.52) | 211 | 0.66 (0.39) | 0.56 (0.35) | 0.74 (0.47) |
| 816 | 0.77 (0.46) | 0.66 (0.41) | 0.57 (0.39) | 254 | 0.81 (0.32) | 0.89 (0.58) | 0.93 (0.62) |
| 851 | 0.87 (0.50) | 0.74 (0.58) | NA | 350 | 0.59 (0.43) | 0.59 (0.37) | 0.56 (0.40) |
| 885 | 0.64 (0.33) | 0.66 (0.40) | 0.40 (0.28) | 374 | 0.47 (0.26) | 0.49 (0.30) | 0.39 (0.22) |
| 894 | 0.67 (0.48) | 0.52 (0.43) | 0.53 (0.32) | 668 | 0.56 (0.42) | 0.71 (0.52) | 0.65 (0.49) |
| 937 | 0.67 (0.51) | 0.28 (0.13) | NA | 682 | 0.60 (0.43) | 0.69 (0.48) | 0.61 (0.40) |
| 1052 | 0.42 (0.27) | 0.43 (0.31) | 0.50 (0.34) | 718 | 1.05 (0.67) | 1.02 (0.62) | NA |
| 1166 | 0.41 (0.23) | 0.42 (0.21) | 0.57 (0.30) | 734 | 0.85 (0.47) | 0.60 (0.48) | 0.61 (0.41) |
| 1180 | 0.73 (0.49) | 0.72 (0.43) | NA | 767 | 0.79 (0.56) | 0.85 (0.70) | 0.69 (0.59) |
| 1480 | 0.52 (0.30) | 0.73 (0.51) | 0.54 (0.33) | 784 | 0.89 (0.50) | 0.79 (0.44) | 0.89 (0.68) |
| 1487 | 0.53 (0.30) | 0.51 (0.36) | 0.63 (0.40) | 792 | 0.60 (0.41) | 0.38 (0.20) | 0.55 (0.36) |
| 1650 | 0.56 (0.20) | 0.62 (0.45) | 0.66 (0.51) | 812 | 0.77 (0.51) | 0.66 (0.44) | 0.58 (0.46) |
| 1681 | 0.69 (0.40) | 0.48 (0.29) | 1.11 (0.87) | 948 | 0.37 (0.13) | 0.74 (0.61) | 0.65 (0.55) |
| 1701 | 0.70 (0.42) | 0.53 (0.40) | 0.42 (0.29) | 1051 | 0.43 (0.18) | 0.55 (0.51) | 0.67 (0.51) |
| 1890 | 0.51 ( 0.22) | 0.45 (0.25) | 0.71 (0.50) | 1130 | 0.71 (0.39) | 0.68 (0.40) | 0.54 (0.32) |
| 2090 | 0.64 (0.45) | 0.58 (0.35) | 0.52 (0.29) | 1335 | 0.52 (0.35) | 0.50 (0.41) | 0.50 (0.46) |
| 2157 | 0.56 (0.31) | 0.50 (0.29) | 0.61 (0.41) | 1466 | 0.83 (0.62) | 0.69 (0.46) | 0.53 (0.40) |
| 2409 | 0.74 (0.39) | 0.63 (0.41) | 0.71 (0.45) | 1802 | 0.78 (0.43) | 0.74 (0.57) | 0.65 (0.46) |
| 3138 | 0.61 (0.41) | 0.51 (0.31) | 0.63 (0.53) | 1848 | 0.64 (0.45) | 0.72 (0.63) | 0.63 (0.53) |
| 3663 | 0.53 (0.24) | 0.53 (0.43) | 0.45 (0.34) | 1981 | 0.50 (0.38) | 0.50 (0.35) | 0.63 (0.39) |
| 3754 | 0.85 (0.49) | 0.56 (0.34) | 0.63 (0.46) | 2233 | 0.32 (0.12) | 0.36 (0.18) | 0.69 (0.47) |
| 3888 | 0.92 (0.70) | 0.69 (0.53) | 0.84 (0.58) | 3002 | 0.41 (0.21) | 0.55 (0.32) | 0.65 (0.25) |
| 4752 | 0.95 (0.16) | 0.59 (0.41) | 0.80 (0.57) | 3393 | 0.82 (0.55) | 0.82 (0.60) | 0.77 (0.55) |
|  |  |  |  | 4833 | 0.52 (0.28) | 0.57 (0.46) | 0.51 (0.48) |
